# Area-Level and Individual-Level Factors for Teenage Motherhood: A Multilevel Analysis in Japan

**DOI:** 10.1371/journal.pone.0166345

**Published:** 2016-11-10

**Authors:** Sachiko Baba, Hiroyasu Iso, Takeo Fujiwara

**Affiliations:** 1 Center for International Relations, Osaka University Graduate School of Medicine, Suita, Japan; 2 Department of Social Medicine, National Research Institute for Child Health and Development, Tokyo, Japan; 3 Public Health, Department of Social Medicine, Osaka University Graduate School of Medicine, Suita, Japan; 4 Department of Global Health Promotion, Tokyo Medical and Dental University, Tokyo, Japan; Hamamatsu Ika Daigaku, JAPAN

## Abstract

**Background:**

Teenage motherhood is strongly associated with a range of disadvantages for both the mother and the child. No epidemiological studies have examined related factors for teenage motherhood at both area and individual levels among Japanese women. Therefore, we performed a multilevel analysis of nationwide data in Japan to explore the association of area- and individual-level factors with teenage motherhood.

**Methods:**

The study population comprised 21,177 mothers living in 47 prefectures who had their first, singleton baby between 10 and 17 January or between 10 and 17 July, 2001. Information on the prefecture in which the mothers resided was linked to prefecture-level variables. Primary outcomes were area-level characteristics (single-mother households, three-generation households, college enrollment, abortions, juvenile crime, and *per capita* income) and individual-level characteristics, and divided into tertiles or quintiles based on their variable distributions. Multilevel logistic regression analysis was then performed.

**Results:**

There were 440 teenage mothers (2.1%) in this study. In addition to individual low level of education [adjusted odds ratio (OR), 7.40; 95% confidence interval (CI), 5.59–9.78], low income [4.23 (2.95–6.08)], and smoking [1.65 (1.31–2.07)], high proportions of single-mother households [1.72 (1.05–2.80)] and three-generation household [1.81 (1.17–2.78)], and *per capita* income [2.19 (1.06–3.81)] at an area level were positively associated, and high level of college enrollment [0.46 (0.25–0.83)] and lower crime rate [0.62 (0.40–0.98)] at area level were inversely associated with teenage motherhood compared with the corresponding women living in prefectures with the lowest levels of these variables.

**Conclusions:**

Our findings suggest that encouraging the completion of higher education and reducing the number of single-mother household at an area level may be important public health strategies to reduce teenage motherhood.

## Introduction

Teenage motherhood is an important issue in terms of maternal and child health [1,2]. For example, teenage motherhood is reported to lead to adverse health outcomes, including coronary heart disease and suicide, due to lower physical and mental health conditions [3–5]. Moreover, teenage motherhood is strongly associated with social deprivation such as school disruption and lower educational attainment [6], unemployment [7], poverty [8], single parenthood [9], and child abuse or neglect [10]. Newborns from teenage pregnancies are associated with low birth weight and preterm delivery [11, 12], which could have a latent effect on the development of diseases during adulthood [13, 14]. Furthermore, children born to teenage mothers are reported to have a variety of adverse health and socioeconomic outcomes and are more likely to develop attention-deficit/hyperactivity disorder [15], to achieve a lower education and personal income [16], and to experience an unsafe and early initiation of sexual intercourse [17, 18]. These children themselves may eventually become teenage parents, starting the cycle all over again [2].

To respond to teenage motherhood, it is important to detect high risk groups and take measures for prevention. Several risk factors for teenage motherhood at individual level have been reported in the US, UK, and Australia, such as a history of childhood abuse, early initiation of sexual activities, being in a single-parent family, being the child of a teenage mother, and initiation of smoking during teenage years [1, 7, 19–22]. However, no epidemiological investigations have been conducted concerning individual related factors for teenage motherhood in Japan or other Asian countries probably because these reported individual factors are very sensitive to be asked about. Another strategy is identifying characteristics at area level where data are regularly collected through existing frameworks as well as at individual level, regardless of causality. This approach may help to reduce the effect of adversity for teenage mothers and their children in these areas.

Some studies have already reported that area-level factors influence teenage motherhood; however, these studies included no individual-level data and were limited to investigating the association of teenage motherhood with social deprivation pertaining to neighborhood levels, which may reflect aggregated individual deprivations [8, 23–25]. Since it is difficult to differentiate the impact of social deprivation at neighborhood level from that at individual level, further studies with both area-level and individual-level factors might be also be helpful.

A Japanese previous study using only area-level variables in Japan showed a strong ecological correlation between early adolescent (i.e., 13–15 years) pregnancy and juvenile delinquency [26]. Based on this finding, we hypothesized that area-level security may have an impact on teenage motherhood. At the same time, we also hypothesized that an area where single-mother households are more common may be associated with higher proportion of teenage motherhood because girls may have more opportunities to consider diverse family structures as their future choices. Similarly, living in a society where three-generation households account for a large proportion of households may provide a socially supportive environment which leads a sense of relief for young women [27, 28].

The purpose of this study was to explore the association of area-level and individual-level factors relating to teenage motherhood, using nationwide data in Japan.

## Materials and Methods

### Study sample

Data for the individual level were obtained from the Japanese panel study entitled “The Longitudinal Survey on Babies Born in the 21^st^ Century.” Based on the birth record lists of Vital Statistics, the study sample included all babies born in Japan between January 10 and 17, 2001, or between July 10 and 17, 2001 (n = 53,575). Selected subjects were recruited by mail questionnaires when the children were 6 months old. The subjects were considered to have agreed to participate in the study if they returned the questionnaire to the Ministry of Health, Labour, and Welfare. There were a total of 47,015 respondents, giving a response rate of 87.8%. Participants who had not provided information on the mother’s age, maternal education, or maternal smoking status were excluded (n = 3,612). Among the remaining 43,403 mothers, those with multiple births and more than one parity, and mothers who did not live with the child were excluded, which lead to a final sample size of 21,159 mothers. A teenage mother was defined as a mother who gave birth when she was less than 20 years old. Mother’s age at delivery was retrieved from the birth record list.

We obtained permission to use these panel data from the Ministry of Health, Labour and Welfare under the Statistics Act in Japan (No.1127-4).

This study was approved by the Ethical Review Board of National Research Institute for Child Health and Development (No.1007).

### Predictor variables

#### Area-level characteristics

Several characteristics of prefectural conditions were taken into consideration. We chose (a) the proportion of single-mother households (%), (b) the proportion of three-generation households (%), (c) the proportion of college enrollment (%), (d) abortion rate (‰), (e) juvenile crime rate (‰), and (f) *per* capita annual income. Proportions of single-mother and three-generation households were obtained from Vital Statistics (special issue) of 2000 [29]. We preferred to use college enrollment rather than high school enrollment because average high school enrollments in Japan are greater than 95%, with very little variance. The proportion of college enrollment is defined as the number of students who enroll in either university or college per 1,000 students who graduate from high school, and was obtained from the School Basic Survey for 2000 [30]. Abortion rate was defined as the number of terminations of pregnancy before 22 weeks’ gestation per 1,000 women aged 15–49 years old [31], and data were obtained from the Report on Public Health Administration and Service for 2000 [32]. Data for *per capita* annual income were obtained from the Annual Report on Prefectural Accounts for fiscal year 2000 [33]. All these data were accessible from the database of the Ministry of Internal Affairs and Communications [34]. The juvenile crime rate was defined as the number of persons aged less than 20 years who committed a crime per 1,000 junior-high and high school students. These data were retrieved from the Report on Minority Protection and Guidance for 2000 published by the National Police Agency [35]. These area-level variables were divided either into tertiles or quintiles based on their distribution, not weighted by the population of prefectures, and linked to individual data according to living prefecture at the time of delivery.

#### Individual-level characteristics

Individual variables used in this study were retrieved from data gathered from the first and the second panel survey. Information on education, couple’s income, cohabitation with the child’s father, and living in a three-generation household were obtained from the second survey (response rate: 93.5%) in 2002. Education was categorized as **“**junior high school**”** or **“**high school or over**”** and couple’s income was categorized as “less than 2 million Japanese Yen” and “2 million Japanese Yen and over”. Cohabitation with the child’s father is categorized as “Yes” if the father live with the family, or father who lives separately but periodically come back to the family, and “No” if the father did not live with for more than 3 months. Information on smoking habits were obtained from questionnaires used in the first survey in 2001, and respondents were classified as nonsmokers or smokers.

### Statistical analyses

Multilevel analyses were performed with 21,159 mothers nested within 47 prefectures. Associations with teenage motherhood were modeled using multilevel logistic regression of the above structure using STATA MP statistical package version 14 (StataCorp L, College Station, Texas, USA). We included similar area-level and individual-level characteristics such as education level, income, and three-generation household to differentiate the impacts of area-level from individual-level characteristics. We first included individual-level variables nested within 47 prefectures (model 1). Next, we added all individual-level and area-level variables nested within the 47 prefectures (model 2). Odds ratios (OR), presented with 95% confidence intervals (CI), were calculated.

## Results

Table 1 presents the correlation coefficients for area-level variables. Among these indicators, college enrollment and *per capita* income were closely correlated. In addition, both were inversely correlated with the proportion of single-mother households and abortion rate.

**Table 1:** Spearman Correlation Coefficients for Area-Level Variables (n = 47).

	Mean	SD	Range	a)	b)	c)	d)	e)	f)
**a) Single-mother households (%)**	6.57	0.89	5.29–10.69	1					
**b) Three-generation households (%)**	13.38	5.72	3.63–28.13	-0.32*	1				
**c) College enrollment (%)**	43.58	7.10	29.90–56.20	-0.49***	-0.23	1			
**d) Abortion rate(‰)**	13.01	3.32	5.60–19.10	0.39**	0.27	-0.58***	1		
**e) Juvenile crime rate (‰)**	12.86	3.27	6.56–20.09	-0.05	-0.10	0.30*	0.05	1	
**f) Per capita annual income (million JPY)**	2.86	0.44	2.11–4.64	-0.70***	-0.04	0.67***	-0.57***	0.21	1

Abbreviation: SD, standard deviation

* *p* <0.05

** *p* <0.01

*** *p* <0.001

Of the 21,159 Japanese women in this study, of those who gave birth to their first, singleton baby between January 10 and 17, 2001 or between July 10 and 17, 2001, 2.1% (440 mothers) were teenagers (Table 2). Among these, 82.6% were 18 or 19 years of age (data not shown). Teenage mothers were more common in prefectures with higher proportions of single-mother households, three-generation households, and lower college enrollment, abortion rates, and lower *per capita* annual incomes. At the individual level, teenage mothers were less-well educated, had lower incomes, and less co-reside with the child’s father (Table 3). Teenage mothers were more likely to reside in a three-generation household, and to smoke.

**Table 2:** Characteristics of the Study Areas and Prevalence of Teenage Motherhood, in Japan, 2001.

Area-level variables (range)	Number of areas	All mothers	Teenage mothers	
		n	n	%
Total	47	21,159	440	2.1
**Single-mother household (%)**				
1 lowest (5.3–6.0)	10	5,058	88	1.7
2 lower (6.0–6.2)	9	6,501	104	1.6
3 middle (6.2–6.6)	10	2,690	81	3.0
4 higher (6.7–7.1)	9	3,197	82	2.6
5 highest (7.2–10.7)	9	3,713	85	2.3
**Three-generation household (%)**				
1 lowest (3.6–8.1)	10	7,679	141	1.8
2 lower (8.2–10.7)	9	6,177	114	1.8
3 middle (11.6–15.1)	10	2,397	56	2.3
4 higher (15.7–18.8)	9	2,979	57	1.9
5 highest (19.0–28.1)	9	1,927	72	3.7
**College enrollment (%)**				
1 low (29.9–40.6)	16	4,278	141	3.3
2 middle (41.5–48.1)	16	7,793	138	1.8
3 high (48.3–56.2)	15	9,088	161	1.8
**Abortion rate (‰)**				
1 low (5.6–11.1)	16	12,463	228	1.8
2 middle (11.3–14.7)	16	4,198	80	1.9
3 high (14.9–19.1)	15	4,498	132	2.9
**Juvenile crime rate (‰)**				
1 lowest (6.6–9.9)	10	4,314	111	2.6
2 lower (10.1–11.7)	9	3,025	49	1.6
3 middle (11.8–13.4)	10	5,585	90	1.6
4 higher (13.5–16.1)	9	1,656	56	3.4
5 highest (16.3–20.1)	9	6,579	134	2.0
**Per capita income (million JPY)**				
1 low (2.1–2.7)	16	3,655	98	2.7
2 middle (2.8–3.0)	16	5,513	130	2.4
3 high (3.0–4.6)	15	11,991	212	1.8

**Table 3:** Characteristics of the Population.

Individual level variable	Total populaition	Teenage mothers	
	n	n	%
**Total**	21,159	440	2.1
**Education**			
Junior-high school	1,135	202	17.8
High school or over	20,024	238	1.2
**Couple's income (JPY)**			
< 2 million	2,027	214	10.6
≥ 2 million	19,132	226	1.2
**Smoking habits**			
Nonsmoker	17,772	255	1.4
Smoker	3,387	185	5.5
**Cohabiting with the child's father**			
Yes	20,429	360	1.8
No	730	80	11.0
**Living in a three-generation household**			
Yes	4,201	227	5.4
No	16,958	213	1.3

The results of the multilevel logistic regression analysis are shown in Table 4. Variances of the random intercept were significantly larger than zero for the null models (results not shown in Tables) and for model 1, while in model 2 (full models), they became zero and non-significant, indicating that no residual area-level variance existed. In model 1, we found that low level of education, low income, smoking, not living with the father, and living in a three-generation household at an individual level were associated with teenage motherhood. In model 2, a higher proportion of single-mother households (Q4), middle and highest proportions of three-generation households (Q3 and Q5), and highest *per capita* income (T3) were associated with teenage motherhood. Also, higher college enrollment rate (T2 and T3) and lower juvenile crime rate (Q2) were inversely associated with teenage motherhood. The abortion rate at an area level was not associated with teenage motherhood.

**Table 4:** Result of Multilevel Analyses for Teenage Motherhood: Adjusted Odds Ratios (and 95% CI).

	Model 1	Model 2	Model 2 for women with higher education (n = 20,024)	Model 2 for women with lower education (n = 1,135)
	Adjusted OR (95%CI)	Adjusted OR (95%CI)	Adjusted OR (95%CI)	Adjusted OR (95%CI)
**Fixed effect**				
**Individual level**				
Education				
Junior-high school	9.39 (7.48–11.80) ***	9.52 (7.56–12.0) ***		
High school or over	Reference	Reference		
Couple's income (JPY)				
< 2 million	5.65 (4.53–7.05) ***	5.67 (4.54–7.08) ***	7.40 (5.59–9.78) ***	4.23 (2.95–6.08) ***
≥ 2 million	Reference	Reference	Reference	1
Smoking habits				
Nonsmoker	Reference	Reference		
Smoker	1.65 (1.31–2.07) ***	1.63 (1.30–2.05) ***		
Cohabiting with the child's father				
Yes	Reference	Reference		
No	1.39 (1.00–1.92) *	1.42 (1.03–1.96) *		
Living in a three-generation household				
Yes	2.95 (2.38–3.65) ***	2.81 (2.26–3.49) ***		
No	Reference	Reference		
**Area level**				
Single-mother household(%)				
Q1 (5.3–6.0)		Reference	Reference	1
Q2 (6.0–6.2)		1.24 (0.9–1.77)	1.44 (0.9–2.27)	0.89 (0.5–1.63)
Q3 (6.2–6.6)		1.37 (0.8–2.27)	1.41 (0.7–2.67)	1.47 (0.7–3.28)
Q4 (6.7–7.1)		1.75 (1.1–2.85) *	1.56 (0.8–2.93)	1.95 (0.9–4.29)
Q5 (7.2–10.7)		1.17 (0.8–1.79)	1.43 (0.8–2.48)	0.91 (0.5–1.74)
Three-generation household (%)				
Q1 (3.6–8.1)		Reference	Reference	1
Q2 (8.2–10.7)		1.38 (0.9–2.05)	1.33 (0.8–2.21)	1.48 (0.8–2.76)
Q3 (11.6–15.1)		1.90 (1.11–3.26) *	2.25 (1.15–4.42) *	1.29 (0.52–3.16)
Q4 (15.7–18.8)		1.24 (0.8–1.93)	1.32 (0.8–2.29)	1.29 (0.6–2.67)
Q5 (19.0–28.1)		1.84 (1.2–2.84) **	2.21 (1.3–3.76) **	1.23 (0.6–2.68)
College enrollment (%)				
T1 (29.9–40.6)		Reference	Reference	1
T2 (41.5–48.1)		0.45 (0.2–0.82) *	0.49 (0.2–1.03)	0.44 (0.2–1.18)
T3 (48.3–56.2)		0.44 (0.2–0.81) *	0.46 (0.2–0.96) *	0.50 (0.2–1.33)
Abortion rate				
T1 (5.6–11.1)		Reference	Reference	1
T2 (11.3–14.7)		1.05 (0.7–1.49)	0.99 (0.6–1.52)	0.97 (0.5–1.76)
T3 (14.9–19.1)		0.95 (0.6–1.45)	0.91 (0.5–1.57)	0.91 (0.5–1.75)
Juvenile crime rate				
Q1 (6.6–9.9)		Reference	Reference	1
Q2 (10.1–11.7)		0.62 (0.39–0.98) *	0.61 (0.35–1.09)	0.63 (0.30–1.32)
Q3 (11.8–13.4)		0.70 (0.5–1.01)	0.75 (0.5–1.19)	0.69 (0.4–1.27)
Q4 (13.5–16.1)		0.95 (0.6–1.49)	1.00 (0.6–1.73)	0.94 (0.4–1.98)
Q5 (16.3–20.1)		1.23 (0.8–1.92)	1.20 (0.7–2.10)	1.40 (0.7–2.90)
Per capita income				
T1 (2.1–2.7)		Reference	Reference	
T2 (2.8–3.0)		1.28 (0.7–2.44)	1.20 (0.5–2.68)	1.17 (0.4–3.34)
T3 (3.0–4.6)		2.30 (1.18–4.46) *	2.05 (0.89–4.73)	2.17 (0.77–6.13) *
**Random effect**				
Area-level variance (standard error)	0.053	0.00	0.00	0.00
sigma_u	0.23	5E-04	5E-04	0.001
p value for variance of random intercept = 0	0.03	1	1	0.496

Adjusted by smoking, cohabitating with the child's father,and living in a three-generation

*p <0.05,

** p <0.01,

***p <0.001

## Discussion

In this study using a nationally representative sample of Japanese mothers, we found that teenage motherhood was associated primarily with individual-level factors, especially low education, but also with area-level factors.

To the best of our knowledge, this is the first study to demonstrate that teenage motherhood is positively associated with high area-level proportions of single-mother households and three-generation households, as well as high *per capita* income, and inversely related to high rates of college enrollment and low juvenile crime rates. We also demonstrated that low level of education and low income at individual level were associated with teenage motherhood; however, this is to be anticipated because teenage birth apparently disrupts schooling, which results in low education and low income.

For area-level characteristics, since an inverse correlation of college enrollment and *per capita* income with proportion of single-mother households were observed, we considered that these indicators may capture the dimension of area deprivation. The proportion of three-generation households has been considered an indicator of social support. Abortion rate has primarily been considered an indicator of unintended pregnancy and attitudes for conforming to be cultural norm, since births outside of marriage represent only 1.7% of all births in Japan [31]. Furthermore, the juvenile crime rate has been considered an indicator of area security.

We found an association of indicators for deprivation (high proportion of single-mother households, low college enrollment) with teenage motherhood, which was accordance with our hypothesis. However, high *per capita* income, high proportions of three-generation households, and low juvenile crime rates, all of which might imply rich social supports, showed a positive association with teenage motherhood. To interpret these findings, we may need to consider that the outcome “teenage motherhood” could be broken down into two elements; teenage pregnancy and choosing to purse pregnancy to birth. In fact, most teenage pregnant women in Japan terminate their pregnancies by induced abortion, with 2.2 abortions for each live birth, in contrast with 0.4 abortions for each live birth in the US [31]. Teenage motherhood might thus reflect a positive decision to avoid induced abortion.

Taking these related factors into account, the effect by high college enrollment (representing least social deprivation) might be inversely associated with the first element (teenage pregnancy) [21, 23, 25] and high proportions of three-generation households, low juvenile crime rates, and a high *per capita* income at an area level might be associated with the latter element (decision to pursuing pregnancy), which may provide teenage pregnant women with their motivation to continue with the pregnancy and ensure child rearing in a safe environment with rich social support [27, 28].

Our results showed that higher proportions of single-mother households were associated with teenage motherhood, except in those areas with the very highest proportions of single-mother households. The mechanism for this effect is unknown. In our study population, 18.1% (80/440) of teenage mothers were not living with their partners (i.e., they were single parents) compared with only 3.5% (730/21159) of mothers of all ages. Since teenage motherhood is a strong predictor for single motherhood [7], we speculate that as the proportion of single-mother households rises, young parenting which might lead to single parenting becomes the social norm in these communities and is considered more acceptable [21, 36]. However, the highest single-mother household areas showed no increased association.

The findings of this study demonstrate the need for implementing strategies for the prevention of teenage motherhood, especially aimed at the prevention of teenage pregnancy, and the following adverse events. Primarily, rising levels of general education is fundamentally important as our findings showed an inverse association with high college enrollment at an area-level [2]. Entry rates into university-level education in Japan ranks 23rd among 34 Organization for Economic Co-operation and Development (OECD) countries, which may partly be due to the fact that public college tuition fees in Japan are the second highest among OECD countries and the public subsidies for higher education, mostly through student loan, but not scholarship [37]. Furthermore, achieving 100% high school enrollment and completion is desirable because 17.8% of teenage mothers did not graduate from high school at an individual level in our study even though the rate for enrollment and completion of high school was generally high (97% and 98%, respectively, in 2000) in Japan [38, 39]. Secondly, robust sex education should be provided both at school and by other local sectors that are accessed outside of the public educational system because more than 80% of teenage mothers were either 18 or 19 years of age and had not achieved completion of high school education. Basic knowledge concerning reproductive rights, including access to contraception, emergency pills, and induced abortion, should be carefully taught, and information on the availability of social welfare such as public assistance should also be provided when teenage pregnancies or other difficult pregnancies arise. Moreover, when these measures result in choosing to pursue a pregnancy, educational and supportive programs for young expectant mothers are essential for the prevention of further unintended pregnancies and for the development of healthy children [40].

Several weaknesses of our study need to be addressed before a conclusion can be achieved. First, the associations of area-level and individual-level variables with teenage motherhood might reflect reverse causation because the temporality is difficult to establish in this study. For example, the association of living in a three-generation household with teenage motherhood might be observed because the young mothers need the support from their parents by living in the same household. However, the characteristics of teenage motherhood both in individual- and area-levels could be captured through this study. Second, a Japanese prefecture, with a population ranging from 0.6 million to 13 million may be too large to determine the effect of area-level factors on individual behavior. A smaller area such as a city may be more appropriate for analysis of the area-level effect. However, the number of teenage mothers in each city may be too small for the analysis of teenage motherhood. In this study, we were able to determine the related factors at a prefecture level, probably because some policies relating to maternal and child health are set at prefecture level as well as at national and municipal levels. Third, information on individual characteristics before pregnancy was limited because the target population for the national survey was determined after delivery. Also, the variable “not living with their partners” may include those whose partner did not live with because of the partner’s job transfer.

## Conclusion

In conclusion, our findings suggest that high proportions of single-mother household, three-generation households, and high *per capita* income were positively associated with teenage motherhood, while high levels of education and low juvenile crime rates were inversely associated. With the aid of panel data, it is essential to confirm whether changes in area-level factors over time may reduce teenage motherhood.
